# Mortality after the 9/11 terrorist attacks among world trade center health registry enrollees with cancer

**DOI:** 10.1002/cam4.4992

**Published:** 2022-09-15

**Authors:** Rebecca D. Kehm, Jiehui Li, Erin Takemoto, Janette Yung, Baozhen Qiao, Mark R. Farfel, James E. Cone

**Affiliations:** ^1^ New York City Department of Health and Mental Hygiene World Trade Center Health Registry Long Island City New York USA; ^2^ Department of Epidemiology Mailman School of Public Health, Columbia University New York City New York USA; ^3^ New York State Department of Health Bureau of Cancer Epidemiology Albany New York USA

**Keywords:** 9/11‐disaster, cancer, mortality, PTSD, trauma

## Abstract

**Background:**

While several studies have reported the association between 9/11 exposure and cancer risk, cancer survival has not been well studied in the World Trade Center (WTC) exposed population. We examined associations of 9/11‐related exposures with mortality in WTC Health Registry enrollees diagnosed with cancer before and after 9/11/2001.

**Patients and Methods:**

This is a longitudinal cohort study of 5061 enrollees with a first‐ever primary invasive cancer diagnosis between 1995 and 2015 and followed through 2016. Based on the timing of first cancer diagnosis, pre‐9/11 (*n* = 634) and post‐9/11 (*n* = 4427) cancer groups were examined separately. 9/11‐related exposures included witnessing traumatic events, injury on 9/11, and 9/11‐related post‐traumatic stress disorder (PTSD). Associations of exposures with all‐cause mortality were examined using Cox proportional hazards regression. In the post‐9/11 group, cancer‐specific mortality was evaluated by enrollee group (WTC rescue/recovery workers vs. non‐workers) using Fine and Gray's proportional sub‐distribution hazard models, adjusting for baseline covariates, tumor characteristics, and treatment.

**Results:**

In the pre‐9/11 group, 9/11‐related exposures were not associated with all‐cause mortality. In the post‐9/11 group, increased risk of all‐cause mortality was associated with PTSD (adjusted HR = 1.35; 95% CI = 1.11–1.65), but not with injury or witnessing traumatic events. Cancer‐specific mortality was not statistically significantly associated with 9/11‐related exposures. In rescue/recovery workers, increased non‐cancer mortality risk was associated with PTSD (aHR = 2.13, 95% CI = 1.13–4.00) and witnessing ≥3 traumatic events (aHR = 2.00, 95% CI = 1.13–3.55).

**Conclusions:**

We did not observe associations between 9/11‐related exposures and cancer‐specific mortality. Similar to findings in the non‐cancer WTC exposed population, PTSD was associated with increased risk of all‐cause mortality in cancer patients.

## Graphical abstract

We observed associations of 9/11‐related exposures with all‐cause mortality, but not with cancer‐specific mortality in individuals diagnosed with cancer after 9/11.

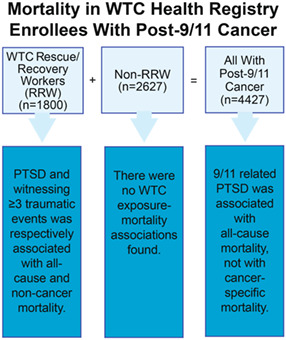

## 1 INTRODUCTION

The World Trade Center (WTC) terrorist attacks on September 11, 2001 in New York City (NYC) killed thousands of people and exposed hundreds of thousands to potentially traumatic events and environmental contaminants that could have long‐lasting health effects. Follow‐up studies support that exposure to the attacks and its aftermath is associated with increased risk of many adverse health conditions, including certain cancers such as prostate, thyroid, and melanoma of the skin.^1^, ^2^, ^3^, ^4^ While several studies have reported on the association between 9/11 exposure and cancer risk, mortality after cancer diagnosis has not been well studied in the WTC‐exposed population.

A recent study compared cancer mortality in WTC‐exposed rescue and recovery workers (RRW) with the general cancer population in New York state and found that all‐cause mortality and cancer‐specific mortality were lower in RRW who were enrolled in one of the federal WTC Health Program's medical monitoring and treatment programs (WTC‐MMTP) but not among those who were not enrolled in the program.^5^ These findings likely reflect the benefits of WTC‐MMTP, including freely available cancer screenings, diagnostic procedures, and treatments for certified 9/11‐related conditions, including cancers, at no cost to patients. Yet, it is unclear if mortality after cancer diagnosis differs in exposed populations (RRW and non‐RRW) by the level of exposure to 9/11‐related events. Previous internal comparisons within the WTC exposed population have demonstrated that the level of exposure on 9/11 has a long‐term impact on health status, where those with the highest levels of exposure consistently have the poorest health outcomes including higher risk of mortality.^6^ These associations might be even more pronounced among those individuals with cancer, given the strong evidence of a link between psychosocial factors (e.g., stress, trauma, post‐traumatic stress disorder (PTSD)) and cancer progression.^7^, ^8^, ^9^

A previous hospital‐based study using trauma registry data from a non‐WTC exposed population found that having a history of trauma is associated with higher cancer‐specific mortality,^7^ and a recent meta‐analysis reported that cancer patients with versus without a pre‐existing mental health condition had a 43% (95% CI, 20–71%) higher risk of cancer‐specific mortality.^8^ These findings suggest that the current understanding of survival time after cancer in the general population may not be applicable to WTC exposed individuals, given the at‐risk health status of this population. The overall health status of the WTC‐exposed population differs from the U.S. general population, in part because multiple WTC‐related chronic health conditions are found to co‐occur in WTC‐exposed individuals.^10^, ^11^, ^12^, ^13^, ^14^, ^15^ This includes long‐term mental health conditions, such as PTSD, stemming from the psychological trauma caused by exposure to the WTC attacks.^16^, ^17^, ^18^ Gaining a better understanding of survival time after cancer in this trauma‐exposed population is thus warranted.

In this study, we used data from the WTC Health Registry (WTCHR) cohort, which enrolled both RRW and non‐RRW, to examine if 9/11‐related exposures are associated with overall mortality and cancer‐specific mortality in individuals diagnosed with cancer. Given that the temporal ordering of traumatic event exposure and cancer diagnosis might impact mortality risk,^7^ we conducted separate analyses for those having the first‐ever primary cancer diagnosis before versus after September 11, 2001. Among those diagnosed after 9/11, we further examined whether associations varied by RRW versus non‐RRW.

## 2 MATERIALS AND METHODS

### 2.1 Study population

The WTCHR is a cohort of individuals exposed to the WTC terrorist attacks (details published elsewhere^19^, ^20^). The cohort includes RRW and volunteers who worked at the WTC site, debris‐loading sites, on barges, or at the Staten Island landfill, and non‐RRW who lived, worked, went to school or were passersby in lower Manhattan, defined as south of Canal Street, on 9/11/2001. Over 71,000 exposed individuals (42.9% RRW, 57.1% non‐RRW) enrolled and completed a baseline survey between 2003–2004.^21^ For this analysis, we included enrollees with a first‐ever primary invasive (or in situ bladder) cancer diagnosis between 1995 and 2015 reported by 10 state cancer registries selected for linkage (New York, New Jersey, California, Connecticut, North Carolina, Massachusetts, Ohio, Pennsylvania, Texas, and Washington) (*n* = 5201). At least 93% of the WTCHR enrollees lived in the catchment areas of these selected registries at some point during follow‐up. All 10 states started their cancer registries in 1995 or earlier and had complete cancer incidence records available through 2015 at the time of linkage. Cancer sites were defined using the Surveillance, Epidemiology, and End Results (SEER) site recode *International Classification for Oncology, Third Edition* (ICD‐O‐3) groups.^22^ We excluded individuals with a first primary cancer diagnosis before age 20 years (*n* = 12), a baseline survey completed by a proxy (*n* = 104), or a missing cancer diagnosis date (*n* = 24). The final sample for this analysis consisted of 5061 enrollees with cancer, which we categorized based on the timing of their first cancer diagnoses as: Pre‐9/11 (*n* = 634) or post‐9/11 (*n* = 4427).

### 2.2 Ascertainment of deaths

Deaths were identified from 2003 (start of WTCHR enrollment) to 2016 through data linkage to the NYC DOHMH Vital Records and the National Death Index. Underlying cause of death was classified using International Classification of Disease codes, 10th revision (ICD‐10). Deaths for which cancer was listed as the underlying cause of death were counted as events in analyses of cancer‐specific mortality; all other deaths were counted as events in analyses of non‐cancer mortality.

### 2.3 9/11‐related exposures

9/11‐related exposures were measured using self‐reported information from the baseline survey, including witnessing traumatic events, physical injuries, and probable PTSD specific to the 9/11 attacks (hereafter referred to as PTSD). We categorized the number of potentially traumatizing events witnessed on 9/11 (seeing an airplane hit one of the WTC towers, a building collapse, people running away from a cloud of smoke, someone injured or killed, people falling or jumping from the towers) as none, 1–2, or ≥3 events. Injuries sustained on 9/11 (yes/no) included cut, abrasion, or puncture wound; sprain or strain; burn; broken bone or dislocation; concussion, head injury, knocked out by being hit on the head. Witnessing traumatic events and physical injuries were selected for analysis because previous research has found them to be significantly associated with 9/11‐related PTSD.^23^

PTSD was evaluated on the baseline survey using the PTSD Checklist‐Specific (PCL‐S), which consists of 17 Likert items (scored 1 = *not at all* to 5 = *extremely*) corresponding to three symptom clusters (re‐experiencing, avoidance, hyperarousal) from the Diagnostic and Statistical Manual of Mental Disorders (DSM‐IV). It is a well‐validated measure with good temporal stability, internal consistency (α > 0.75), test‐retest reliability (correlation coefficient *r* = 0.66), and high convergent validity (*r* = 0.58–0.93).^24^, ^25^ This instrument was used to evaluate the extent to which 9/11 event‐specific symptoms bothered respondents in the past 4 weeks. We defined PTSD as a PCL‐S score of ≥50 to increase specificity and minimize false positives.^26^

### 2.4 Covariates

Baseline covariates were selected a priori following literature review and included self‐reported age on 9/11/2001, gender, socioeconomic status (SES; based on education attainment and household income), race/ethnicity, smoking history, pre‐9/11 physical and mental health conditions, and enrollee group. Enrollees were grouped as RRW or non‐RRW. Non‐RRW included residents, area workers, passersby, local school staff, and students. We derived a count (0, 1, 2, ≥3) of pre‐9/11 physical health conditions, including asthma, hypertension, heart condition (angina, myocardial infarction, heart disease, or other heart condition), stroke, emphysema, and diabetes. Pre‐9/11 mental health symptoms included depression, anxiety, or other emotional problems, but not PTSD.

### 2.5 Tumor and prognostic characteristics

Variables related to the first cancer diagnosis included age at diagnosis, diagnosis year, cancer site, stage at diagnosis, grade, type of initial course of treatment (surgery, radiation, chemotherapy), and subsequent cancer diagnoses (any/none). To account for cancer types that have better prognoses and are more amenable to treatment than others, we categorized first cancers by their 5‐year relative survival rates based on SEER18 data^27^ and previously defined cut points.^28^

### 2.6 Statistical analyses

All analyses were conducted separately for the pre‐9/11 and post‐9/11 groups because of the differences in the temporal ordering of events, which has been shown to impact mortality risk in previous research.^7^ There were also major differences between the two groups in terms of baseline factors and tumor characteristics (see Tables 1 and 2), which likely reflect the fact that the pre‐9/11 group had the added requirement of surviving their cancer diagnosis long enough to be included in the cohort. Furthermore, cancer treatment data were not systematically collected by some cancer registries until 2004 (e.g., NYS), and thus treatment adjusted models could only be evaluated in the post‐9/11 group. Given these differences, the pre‐9/11 and post‐9/11 groups were not directly compared as part of this analysis.

**TABLE 1:** Baseline characteristics of enrollees diagnosed with cancer in the World Trade Center Health Registry, stratified by timing of first cancer diagnosis relative to 9/11/2001

	Timing of first cancer diagnosis		
	Pre‐9/11	Post‐9/11	
Characteristic at enrollment, *N* (%)	(*n* = 634)	(*n* = 4427)	*p*‐value^a^
Age on 9/11/2001, years			
<25	<5 (0.2)	48 (1.1)	<0.001
25–44	153 (24.1)	1318 (29.8)	
45–64	351 (55.4)	2703 (61.1)	
≥65	129 (20.4)	358 (8.1)	
Rescue/recovery worker			
Yes	195 (30.8)	1800 (40.7)	<0.001
No^b^	439 (69.2)	2627 (59.3)	
Gender			
Female	285 (45.0)	1643 (37.1)	<0.001
Male	349 (55.1)	2784 (62.9)	
Race and ethnicity			
Non‐Hispanic White	446 (70.4)	3104 (70.1)	0.15
Non‐Hispanic Black	77 (12.2)	654 (14.8)	
Non‐Hispanic, other race	52 (8.2)	287 (6.5)	
Hispanic	59 (9.3)	382 (8.6)	
Socioeconomic status^c^			
Low	179 (28.2)	1063 (24.0)	0.004
Moderate	132 (20.8)	1035 (23.4)	
High	220 (34.7)	1751 (39.6)	
Unknown	103 (16.3)	578 (13.1)	
Smoking status			
Never	316 (49.8)	2098 (47.4)	0.05
Former	234 (36.9)	1581 (35.7)	
Current	75 (11.8)	687 (15.5)	
Unknown	9 (1.4)	61 (1.4)	
Pre‐9/11 physical health conditions (excluding cancer)			
None	386 (60.9)	2868 (64.8)	0.01
1 condition	169 (26.7)	1166 (26.3)	
2+ conditions	79 (12.5)	393 (8.9)	
Pre‐9/11 mental disorder symptoms			
No	561 (88.5)	4001 (90.4)	0.32
Yes	65 (10.3)	377 (8.5)	
Unknown	8 (1.3)	49 (1.1)	
Traumatic events witnessed on 9/11			
None	192 (30.3)	1235 (27.9)	0.35
1–2 events	203 (32.0)	1477 (33.4)	
≥3 events	216 (34.1)	1607 (36.3)	
Unknown	23 (3.6)	108 (2.4)	
Injury sustained on 9/11			
No	565 (89.1)	3781 (85.4)	0.01
Yes	63 (9.9)	611 (13.8)	
Unknown	6 (0.9)	35 (0.8)	
PTSD related to 9/11			
No	552 (87.1)	3845 (86.9)	0.72
Yes	71 (11.2)	519 (11.7)	
Unknown	11 (1.7)	63 (1.4)	

Abbreviation: PTSD, post‐traumatic stress disorder.

^a^ *p*‐value is reported from a 2‐sided Pearson Chi‐squared test.

^b^ Include residents, area workers, passersby, and school staff and students.

^c^ A 3‐level socioeconomic status (SES) variable was derived using baseline household income and educational attainment data. Low SES is defined as a household income of <$50,000 per year and a college degree or less. Medium SES is defined as a household income of <$50,000 per year and more that a college degree; or a household income of $50,000–$149,999 per year and a high school degree or less. High SES is defined as a household income of $75,000–$149,999 per year and a college degree or more; a household income of ≥$50,000 per year and more than a college degree; or a household income of ≥$150,000 and any education. These categorizations were selected based on the distribution of the sample.

**TABLE 2:** Prognostic characteristics of the first cancer in enrollees in the World Trade Center Health Registry, stratified by timing of first cancer diagnosis relative to 9/11/2001

Vital status and characteristic of first cancer, *N* (%)	Timing of first cancer diagnosis		*p*‐value^a^
	Pre‐9/11	Post‐9/11	
	(*n* = 634)	(*n* = 4427)	
Vital status			
Alive	498 (78.5)	3361 (75.9)	<0.001
Deceased, cancer‐related cause of death	83 (13.1)	868 (19.6)	
Deceased, non‐cancer cause of death	53 (8.4)	198 (4.5)	
Age at diagnosis			
<40 years	125 (19.7)	268 (6.1)	<0.001
40–49 years	151 (23.8)	754 (17.0)	
50–59 years	209 (33.0)	1520 (34.3)	
60–69 years	95 (15.0)	1307 (29.5)	
70–79 years	42 (6.6)	436 (9.9)	
80+ years	12 (1.9)	142 (3.2)	
Year of diagnosis			
1995–2005	634 (100.0)	1036 (23.4)	<0.001
2006–2010	0 (0.0)	1585 (35.8)	
2010–2015	0 (0.0)	1806 (40.8)	
Subsequent cancer diagnoses after first cancer			
Yes	130 (20.5)	350 (7.9)	<0.001
No	504 (79.5)	4077 (92.1)	
5‐year relative survival rate^b^			
<40%	36 (5.7)	792 (17.9)	<0.001
40–69%	110 (17.4)	814 (18.4)	
≥70%	488 (77.0)	2821 (63.7)	
Cancer site^c^			
Prostate	129 (20.4)	1017 (23.0)	<0.001
Breast	148 (23.3)	608 (13.7)	
Lung and bronchus	15 (2.4)	316 (7.1)	
Thyroid	36 (5.7)	225 (5.1)	
Skin melanomas	38 (6.0)	204 (4.6)	
Kidney and renal pelvis	24 (3.8)	174 (3.9)	
Bladder	21 (3.3)	151 (3.4)	
Non‐Hodgkin's lymphomas nodal	15 (2.4)	142 (3.2)	
Corpus uteri	26 (4.1)	132 (3.0)	
Non‐Hodgkin's lymphomas extranodal	12 (1.9)	87 (2.0)	
Others	170 (26.8)	1371 (31.0)	
Stage at diagnosis			
Localized	396 (62.5)	2482 (56.1)	<0.001
Regional	133 (21.0)	899 (20.3)	
Distant	43 (6.8)	790 (17.9)	
Unknown	62 (9.8)	256 (5.8)	
Grade			
Well differentiated	64 (10.1)	430 (9.7)	<0.001
Moderately differentiated	215 (33.9)	1229 (27.8)	
Poorly differentiated	111 (17.5)	1033 (23.3)	
Undifferentiated	5 (0.8)	119 (2.7)	
Other (V–VIII)	24 (3.8)	330 (7.5)	
Unknown	215 (33.9)	1286 (29.1)	
Receipt of surgery^d^			
Yes	80 (12.6)	2988 (67.5)	<0.001
No	30 (4.7)	1214 (27.4)	
Unknown	524 (82.7)	225 (5.1)	
Receipt of radiation^d^			
Yes	22 (3.5)	1245 (28.1)	<0.001
No	6 (1.0)	2216 (50.1)	
Unknown	606 (95.6)	966 (21.8)	
Receipt of chemotherapy^d^			
Yes	17 (2.7)	1253 (28.3)	<0.001
No	81 (12.8)	2720 (61.4)	
Unknown	536 (84.5)	454 (10.3)	

^a^ *p*‐value is reported from a 2‐sided Pearson Chi‐square test.

^b^ 5‐year relative survival rate for each cancer type calculated based on Surveillance Epidemiology and End Results 18 data.

^c^ Based on the 10 most common cancer sites diagnosed in the post‐9/11 group.

^d^ Cancer treatment data were not systematically collected by some cancer registries until 2004 (e.g., NYS).

In both cancer groups, we used Cox proportional hazards (PH) regression to calculate adjusted hazard ratios (aHRs) and 95% confidence intervals (CIs) for all‐cause mortality. Each of the three 9/11‐related exposures was examined in a separate multivariable model. Person‐time from first cancer diagnosis to death or censoring (12/31/2016) was used as the underlying time scale. Follow‐up time was left truncated at the date of baseline survey in those diagnosed with cancer before study enrollment to avoid potential survival bias. We examined models that were stratified by age group at first cancer diagnosis and adjusted for baseline covariates, prognostic characteristics of the first cancer (year, stage, grade, and 5‐year relative survival rate), and subsequent cancer diagnoses (any/none). Indicator variables were used to account for missing covariate data, while observations with missing WTC‐exposure data were excluded from analyses. We conducted a complete case analysis as a sensitivity analysis, substituting education for the derived SES variable to reduce the number of excluded cases.

In the post‐9/11 group, we examined models further adjusted for initial cancer treatment and conducted a competing risks analysis to calculate aHRs and 95% CIs for cancer‐specific mortality (non‐cancer deaths counted as competing events) and non‐cancer mortality (cancer deaths counted as competing events) using Fine and Gray's proportional subdistribution hazard models.^29^ We evaluated whether the associations differed by enrollee group (RRW vs. non‐RRW) by including a cross‐product term between exposure and enrollee group in models; the Wald test statistic was used to determine statistical significance. We also evaluated whether associations differed by gender or cancer subtype prognosis (3‐level variable based on 5‐year survival rates) but found no evidence of effect modification by these factors; these cross‐product terms were excluded from the final models. We assessed the PH assumption with Schoenfeld residuals methods.^30^ The PH assumption did not hold for pre‐9/11 physical conditions in the post‐9/11 group and so we included an interaction term between this covariate and time in models.

We conducted sensitivity analyses in the post‐9/11 group by repeating the fully adjusted model for the association between 9/11‐related exposure and mortality in the following subgroups: (1) Excluding those aged ≥65 years on 9/11/2001 to capture potential premature mortality; (2) excluding firefighters and police responders affiliated with the Fire Department of New York City (FDNY) and the New York City Police Department (NYPD), respectively, because previous research suggests lower rates of PTSD^31^ and higher survival^5^ in traditional versus non‐traditional responders (defined elsewhere^32^); and (3) excluding those with multiple cancers because subsequent cancer may have a worse survival rate than the first. Statistical analyses were performed using SAS, v9.4 (SAS Institute Inc.). All statistical tests were 2‐sided and *p* values <0.05 were considered statistically significant.

## 3 RESULTS

### 3.1 Pre‐9/11 group

Information about the baseline characteristics of the sample is provided in Table 1, while information about the first cancer is provided in Table 2. In the pre‐9/11 group, median person‐time (interquartile range) from first cancer diagnosis to death or censoring was 16.9 (3.6) years. Median person‐time (interquartile range) from first cancer diagnosis to study enrollment was 5.3 (3.2) years. Over follow‐up, there were 136 deaths, 61.0% of which were cancer‐specific deaths. No associations were found between any of the 9/11‐related exposures and all‐cause mortality in the pre‐9/11 group (Table 3).

**TABLE 3:** Adjusted hazard ratios of all‐cause mortality by 9/11‐related exposures and PTSD symptoms in the World Trade Center Health Registry, stratified by timing of first cancer diagnosis relative to 9/11/2001

	Exposure levels being compared	Timing of first cancer diagnosis	
		Pre‐9/11	Post‐9/11
		(*n* = 634)	(*n* = 4427)
Exposure		AHR (95% CI)^a^	AHR (95% CI)^b^
9/11‐related PTSD	yes vs. no	0.83 (0.41, 1.67)	1.35 (1.11, 1.65)
Injury sustained on 9/11	yes vs. no	0.94 (0.50, 1.77)	1.04 (0.86, 1.27)
Number of traumatic events witnessed on 9/11	1–2 vs. none	1.02 (0.58, 1.78)	1.02 (0.84, 1.24)
	≥3 vs. none	1.53 (0.91, 2.59)	1.12 (0.93, 1.35)

Abbreviations: AHR, adjusted hazard ratio; CI, confidence interval; PTSD, post‐traumatic stress disorder.

^a^ Model is stratified by age at first cancer diagnosis in 10‐year intervals and adjusted for socioeconomic status, gender, race/ethnicity, enrollee group, smoking status, pre‐9/11 mental health disorder symptoms, pre‐9/11 physical health conditions, year of diagnosis (continuous), 5‐year relative survival rate of first cancer type, stage at diagnosis of first cancer, grade of first cancer, and any subsequent cancer diagnoses after first cancer.

^b^ Model is stratified by age at first cancer diagnosis in 10‐year intervals and adjusted for socioeconomic status, gender, race/ethnicity, enrollee group, smoking status, pre‐9/11 mental health disorder symptoms, pre‐9/11 physical health conditions, interaction term between pre‐9/11 physical health conditions and time, year of diagnosis (continuous), 5‐year relative survival rate of first cancer type, stage at diagnosis of first cancer, grade of first cancer, and any subsequent cancer diagnoses after first cancer.

### 3.2 Post‐9/11 group

In the post‐9/11 group, median person‐time (interquartile range) from first cancer diagnosis to death or censoring was 5.5 (6.9) years. Fourteen percent of the post‐9/11 group was diagnosed with first cancer between 9/11/2001 and study enrollment. Over follow‐up, there were 1066 deaths, 81.4% of which were cancer‐specific deaths. PTSD was statistically significantly associated with 35% higher risk of all‐cause mortality in the post‐9/11 group after adjusting for baseline covariates and tumor characteristics (aHR = 1.35, 95% CI = 1.11–1.65). After further adjustment for initial course of treatment (Table 4), PTSD was statistically significantly associated with 27% higher risk of all‐cause mortality (aHR = 1.27, 95% CI = 1.05–1.55), but not with cancer‐specific mortality (aHR = 1.17, 95% CI = 0.93–1.46) or non‐cancer mortality (aHR = 1.50, 95% CI = 0.99–2.27). Overall, injury and witnessing traumatic events on 9/11 were not associated with mortality in the post‐9/11 group. In RRW, PTSD and witnessing ≥3 traumatic events (versus none) were statistically significantly associated with all‐cause mortality (aHR = 1.57, 95% CI = 1.16–2.12 and aHR = 1.28, 95% CI = 1.00–1.64, respectively) and non‐cancer mortality (aHR = 2.13, 95% CI = 1.13–4.00 and aHR = 2.00, 95% CI = 1.13–3.55, respectively). However, there was no statistical evidence of effect modification by enrollee group (RRW vs. non‐RRW; all cross‐product term *p*‐values >0.05).

**TABLE 4:** Treatment‐adjusted hazard ratios of all‐cause, cancer‐specific, and non‐cancer mortality by 9/11‐related exposures, overall and including interaction terms by enrollee group, in enrollees first diagnosed with cancer after 9/11/2001 in the World Trade Center Health Registry (*n* = 4427)^a^

Outcome	Exposure	Exposure levels being compared		Enrollee group^b^		
			All	RRW	Non‐RRW	*p*‐value
			AHR (95% CI)	AHR (95% CI)	AHR (95% CI)	
All‐cause mortality	9/11‐related PTSD	yes vs. no	1.27 (1.05, 1.55)	1.57 (1.16, 2.12)	1.17 (0.92, 1.48)	0.11
	Injury sustained on 9/11	yes vs. no	1.06 (0.87, 1.28)	1.02 (0.75, 1.38)	1.08 (0.84, 1.39)	0.77
	Number of traumatic events witnessed on 9/11	1–2 vs. none	0.99 (0.81, 1.20)	1.10 (0.82, 1.48)	0.84 (0.65, 1.10)	0.18
		≥3 vs. none	1.08 (0.90, 1.30)	1.28 (1.00, 1.64)	0.90 (0.69, 1.17)	0.05
Cancer‐specific mortality^c^	9/11‐related PTSD	yes vs. no	1.17 (0.93, 1.46)	1.25 (0.85, 1.82)	1.14 (0.88, 1.47)	0.69
	Injury sustained on 9/11	yes vs. no	0.99 (0.80, 1.23)	0.96 (0.67, 1.37)	1.02 (0.77, 1.33)	0.80
	Number of traumatic events witnessed on 9/11	1–2 vs. none	0.94 (0.76, 1.15)	0.99 (0.72, 1.35)	0.87 (0.66, 1.16)	0.58
		≥3 vs. none	0.99 (0.81, 1.22)	1.08 (0.81, 1.44)	0.91 (0.68, 1.21)	0.43
Non‐cancer mortality^c^	9/11‐related PTSD	yes vs. no	1.50 (0.99, 2.27)	2.13 (1.13, 4.00)	1.25 (0.75, 2.10)	0.19
	Injury sustained on 9/11	yes vs. no	1.22 (0.80, 1.86)	1.25 (0.63, 2.46)	1.20 (0.71, 2.01)	0.92
	Number of traumatic events witnessed on 9/11	1–2 vs. none	1.17 (0.77, 1.78)	1.32 (0.69, 2.55)	0.93 (0.55, 1.56)	0.17
		≥3 vs. none	1.35 (0.88, 2.06)	2.00 (1.13, 3.55)	0.96 (0.56, 1.64)	0.06

Abbreviations: AHR, adjusted hazard ratio; CI, confidence interval; PTSD, post‐traumatic stress disorder; RRW, WTC rescue and recovery workers.

^a^ Cox proportional hazards model is stratified by age at first cancer diagnosis in 10‐year intervals and adjusted for socioeconomic status, gender, race/ethnicity, enrollee group, smoking status, pre‐9/11 mental health disorder symptoms, pre‐9/11 physical health conditions, interaction term between pre‐9/11 physical health conditions and time, year of diagnosis (continuous), 5‐year relative survival rate of first cancer type, stage at diagnosis of first cancer, grade of first cancer, any subsequent cancer diagnoses after first cancer, and initial course of treatment for first cancer (surgery, radiation, and chemotherapy). A cross‐product term between exposure and enrollee group is also included in the model predicting enrollee‐specific estimates.

^b^ *p*‐value is reported from the Wald test statistic for the interaction term between the exposure variable and enrollee group (RRW vs. non‐RRW).

^c^ Competing risks model is stratified by age at first cancer diagnosis in 10‐year intervals and adjusted for the same covariates and cross‐product terms as the Cox proportional hazards model. Deaths due to other causes are counted as competing events.

Results from the complete case analysis were consistent with the main findings. Sensitivity analyses in the post‐9/11 group excluding those ≥65 years on 9/11/2001 and those with subsequent cancers were consistent with the main findings. Associations were generally consistent with main findings when FDNY and NYPD responders (*n* = 399, 22.2% of RRW) were excluded from the sample, except that the association between PTSD and cancer‐specific mortality was larger in magnitude and statistically significant (aHR = 1.55, 95% CI = 1.09–2.20, Table S1).

## 4 DISCUSSION

This is the first study to evaluate 9/11‐related exposures in association with all‐cause, cancer‐specific, and non‐cancer mortality in RRW and non‐RRW diagnosed with cancer in the WTCHR. We found no associations between the 9/11‐related exposures and all‐cause mortality in the pre‐9/11 group. In the post‐9/11 group, we found an overall association between 9/11‐related PTSD and higher risk of all‐cause mortality. Injury sustained on 9/11 has been reported to be one of the strongest predictors of post‐9/11 PTSD^23^ and significantly associated with increased report of post‐9/11 chronic disease, even in the absence of probable PTSD.^33^ However, we observed no association of injury with mortality risk in the post‐9/11 group, which suggests that the impact of injury on survival in cancer patients may be minimal or need longer follow‐up to determine. We also did not find an overall association between witnessing traumatic events and all‐cause mortality in the post‐9/11 group. When we examined cause‐specific mortality in the post‐9/11 group, we found no overall associations between the 9/11‐related exposures and cancer‐specific or non‐cancer mortality.

Our finding that 9/11‐related PTSD is associated with increased risk of all‐cause mortality in the post‐9/11 cancer group is consistent with previous mortality studies of WTCHR enrollees. For example, a previous study using the full cohort (all individuals irrespective of cancer history) found that 9/11‐related exposure levels were associated with 40% increased risk of all‐cause mortality in RRW, but found weaker associations in non‐RRW.^6^ Another study found that 9/11‐related PTSD was associated with 63% increased risk of all‐cause mortality in RRW and 38% in non‐RRW.^34^ It is reasonable that the same mechanisms that are suspected to increase mortality in individuals with PTSD in the cancer‐free population are also relevant to the cancer population. This includes behavioral factors associated with PTSD, such as tobacco smoking, as well as biological mechanisms including elevated basal heart rate, systemic inflammation, cellular dysfunction, and neuroendocrine activation.^35^, ^36^, ^37^ The lack of an association between PTSD and all‐cause mortality in the pre‐9/11 group might be due to small sample size or other factors that differed between the cancer groups such as the timing of trauma relative to first cancer diagnosis. A previous hospital‐based study using trauma registry data from a non‐WTC‐exposed population also found that the timing of trauma relative to cancer diagnosis was important for mortality risk, with individuals who experienced a traumatic episode before versus after cancer diagnosis having a 4.6‐fold increased risk of death.^7^ Taken together, these findings support the need for further studies that are focused on elucidating the biological mechanisms through which a history of trauma impacts mortality risk in patients newly diagnosed with cancer.

Longer follow‐up time might be needed to observe an association with cancer‐specific mortality, especially given that over 60% of cancers in the post‐9/11 group had a ≥70% 5‐year survival rate. The availability of the WTC Health Program at no out‐of‐pocket cost to enrollees also might have impacted cancer‐specific mortality. Being enrolled in the WTC Health Program has been associated with lower cancer‐specific mortality in RRW,^5^ and individuals with self‐reported 9/11‐related physical or mental health conditions have been targeted in outreach efforts to increase WTC Health Program enrollment.^38^

When we stratified the post‐9/11 group by enrollee group, we observed that witnessing ≥3 traumatic events was associated with a 2‐fold increased risk in RRW, which was similar in magnitude to the association between PTSD and non‐cancer mortality in this enrollee group and aligns with previous research in the WTCHR that has found witnessing traumatic events on 9/11 to be a major predictor of PTSD.^23^ While we did not find statistical evidence of a multiplicative interaction when we formally tested for effect modification by enrollee group, these associations are worth discussing given the magnitude and direction of the associations and the consistency with prior studies. Additional studies with larger samples and longer follow‐up time are needed to further explore how PTSD might impact mortality in RRW diagnosed with cancer.

Limitations are noted. We were limited by the use of self‐reported exposures and covariate data obtained from baseline surveys administered 2–3 years after 9/11/2001, which might be subject to recall bias. Furthermore, some variables in our analysis could change over time (e.g., PTSD, SES), which means that baseline measures might not accurately reflect measures from around the time of outcome. A previous WTCHR suggests that our analysis of baseline PTSD might have led to more conservative estimates than if we had used a time‐varying measure of PTSD.^34^ Thus, the significant association of PTSD with all‐cause mortality may have been underestimated. Additionally, our PTSD measure was not based on a clinical diagnosis, although the PCL‐S is shown to correlate highly with clinician‐administered measures,^25^ and we used a score of 50 as our cut‐off to minimize the overestimation of PTSD. In analyses, we did not evaluate post‐9/11 depression or other mental disorders, which often co‐occur with PTSD and can cause collider bias if not properly accounted for in the study design or analysis.^39^ This study was also limited by the presence of missing data, which could have biased results. We excluded observations with missing WTC exposure data, and findings were consistent regardless of whether missing covariate data were modeled using indicator variables or excluded (i.e., complete case analysis). Further, we did not account for the tumor characteristics of subsequent cancers. However, sensitivity analysis findings were consistent with the main analysis when we restricted the post‐9/11 group to individuals without subsequent cancer. Finally, we did not adjust for multiple comparisons, which would have increased type II error,^40^ and therefore some of the statistically significant findings could be due to chance. However, the consistency of in the main analysis and sensitivity analyses supports that our findings are not merely spurious associations. The limitations of this study are offset by several strengths, including that this is the first study to evaluate mortality by level of 9/11‐related exposures in RRW and non‐RRW with cancer, consideration of all‐cause and cause‐specific mortality using competing risks models, and examination of distinct subgroups of individuals affected by cancer before and after the traumatic events on 9/11/2001.

## 5 CONCLUSION

We did not observe associations between 9/11‐related exposures and cancer‐specific mortality in this study, which might require longer follow‐up. Similar to findings in the non‐cancer WTC exposed population, cancer patients with PTSD were found to be at increased risk of all‐cause mortality. This supports the need for enhanced screening for underlying mental health symptoms in individuals newly diagnosed with cancer in the WTCHR, as well as services that can mitigate the risk of death in this population. More broadly, this finding supports the need for continued research on the long‐term mental health effects of trauma in cancer patients.

## AUTHOR CONTRIBUTIONS

Rebecca D Kehm: Conceptualization, Formal Analysis, Methodology, Validation, Visualization, Writing – original draft, Writing – review & editing. Jiehui Li: Conceptualization, Data curation, Investigation, Formal Analysis, Methodology, Project administration, Validation, Visualization, Writing – review & editing. Erin Takemoto: Methodology, Visualization, Writing – review & editing. Janette Yung: Data curation, Validation, Visualization, Writing – review & editing. Baozhen Qiao: Methodology, Writing – review & editing. Mark R Farfel: Funding acquisition, Resources, Supervision, Writing – review & editing. James E. Cone: Conceptualization, Funding acquisition, Project administration, Supervision, Writing – review & editing.

## FUNDING INFORMATION

National Institute for Occupational Safety and Health of U.S. CDC: 2U50/OH009739 and 5U50/OH009739 New York City Department of Health and Mental Hygiene New York State Department of Heath

## CONFLICT OF INTEREST

The authors declare no conflicts of interest.

## DISCLAIMERS

Its contents are solely the responsibility of the authors and do not necessarily represent the official views of the Centers for Disease Control and Prevention—National Institute for Occupational Safety and Health. There are additional disclaimers specified by individual State Cancer Registry: “The ideas and opinions expressed herein are those of the author(s) and do not necessarily reflect the opinions of the State of California, Department of Public Health, the National Cancer Institute, and the Centers for Disease Control and Prevention or their Contractors and Subcontractors.” “The Connecticut Department of Public Health does not endorse or assume any responsibility for any analyses, interpretations or conclusions based on the data. The authors assume full responsibility for all such analyses, interpretations and conclusions.” “The views expressed herein are solely those of the author(s) and not necessarily reflect those of the Florida Department of Health (DOH) or the Centers for Disease Control and Prevention, National Program of Cancer Registries (CDC‐NPCR).” “Use of these data does not imply that Ohio Department of Health (ODH) or the Centers for Disease Control and Prevention (CDC) agrees or disagrees with the analyses, interpretations or conclusions in this publication.” “The Pennsylvania Department of Health specifically disclaims responsibility for any analyses, interpretations or conclusions.”

## ETHICS APPROVAL AND CONSENT TO PARTICIPATE

This study was approved by the Institutional Review Board (IRB) of the NYC Department of Health and Mental Hygiene (DOHMH). Each cancer registry record linkage was also approved by the respective IRB of 10 state departments of health and the Rutgers University of New Jersey. A Federal Certificate of Confidentiality was obtained, and all participants provided verbal informed consent for their responses to be used in data linkages and analyses.

## ROLE OF THE FUNDERS

The funders of the study had no role in design and conduct of the study, analysis, interpretation of the data, or decision to submit the manuscript for publication.

## Supporting information


Appendix S1

## Data Availability

World Trade Center Health Registry data may be made available following review of applications to the Registry from external researchers. Cancer data from 10 state cancer registries may be requested from those entities separately. The data are not publicly available due to privacy or ethical restrictions.
