# Supplementary material for: Mortality after the 9/11 terrorist attacks among world trade center health registry enrollees with cancer
Source: Cancer Med. 2022 Sep 15;12(2):1829–40. doi: 10.1002/cam4.4992 (PMC9883583; doi:10.1002/cam4.4992)
Supplement: Supplementary file 1 — Appendix S1 [file CAM4-12-1829-s001.docx]

**Mortality after the 9/11 terrorist attacks among World Trade Center Health Registry enrollees with cancer**

Rebecca D Kehm, PhD^a,b^, Jiehui Li, MBBS, MS^a^, Erin Takemoto, PhD^a^, Janette Yung, MPH^a^, Baozhen Qiao, PhD^c^ , Mark R Farfel, ScD^a^ James E. Cone, MD, MPH^a^

Supplementary Table. Sensitivity analysis by repeating models in Table 4 for the association between 9/11-related exposure and mortality in the post-9/11 group, excluding those affiliated with FDNY and NYPD (N=4,028)

|  | Exposure levels  being compared | All-cause mortality | Cancer-specific mortality | Non-cancer mortality |  |
| --- | --- | --- | --- | --- | --- |
| Exposure |  | AHR (95% CI)^a^ | AHR (95% CI)^b^ | AHR (95% CI)^b^ |  |
| 9/11-related PTSD | yes vs. no | 1.65 (1.19-2.29) | 1.55 (1.09-2.20) | 1.66 (0.81-3.42) |  |
| Injury sustained on 9/11 | yes vs. no | 1.03 (0.70-1.50) | 1.07 (0.71-1.61) | 1.10 (0.47-2.57) |  |
| Witnessing traumatic  events on 9/11 | 1-2 vs. none | 1.18 (0.86-1.62) | 1.00 (0.71-1.41) | 1.59 (0.81-3.11) |  |
|  | ≥3 vs. none | 1.14 (0.86-1.51) | 0.97 (0.71-1.35) | 1.87 (0.99-3.55) |  |
|  |  |  |  |  |  |
| Abbreviations: CI = Confidence Interval; AHR = Adjusted Hazard Ratio; PTSD = post-traumatic stress disorder. | | | | | |
| ^a^Cox proportional hazards model is stratified by age group at first cancer diagnosis and adjusted  for socioeconomic status, gender, race/ethnicity, enrollee type, smoking status, pre-9/11 mental health disorder symptoms, pre-9/11 physical health conditions, interaction term between pre-9/11 physical health conditions and time, year of diagnosis (continuous), 5-year relative survival rate of first cancer type, stage at diagnosis of first cancer, grade of first cancer, any subsequent cancer diagnoses after first cancer, and initial course of treatment for first cancer (surgery, radiation, and chemotherapy). | | | | | |
| ^b^Competing risks model is stratified by age group at first cancer diagnosis and adjusted  for socioeconomic status, gender, race/ethnicity, enrollee type, smoking status, pre-9/11 mental health disorder symptoms, pre-9/11 physical health conditions, interaction term between pre-9/11 physical health conditions and time, year of diagnosis (continuous), 5-year relative survival rate of first cancer type, stage at diagnosis of first cancer, grade of first cancer, any subsequent cancer diagnoses after first cancer, and initial course of treatment for first cancer (surgery, radiation, and chemotherapy). | | | | | |
